# Strategies to Maximize the Benefits of Evidence-Based Enteral Nutrition: A Narrative Review

**DOI:** 10.3390/nu17050845

**Published:** 2025-02-28

**Authors:** Ken-Ichi Kano, Ryo Yamamoto, Minoru Yoshida, Takeaki Sato, Yoshihiro Nishita, Jiro Ito, Kazuki Nagatomo, Hiroyuki Ohbe, Kanako Takahashi, Masayuki Kaku, Hideaki Sakuramoto, Nobuto Nakanishi, Kazushige Inoue, Junji Hatakeyama, Hidenori Kasuya, Minoru Hayashi, Takefumi Tsunemitsu, Hiroomi Tatsumi, Naoki Higashibeppu, Kensuke Nakamura

**Affiliations:** 1 Department of Pharmacoepidemiology, Graduate School of Medicine, Kyoto University, Kyoto 606-8501, Japan; kanoken.1023.182.d@gmail.com; 2 Department of Emergency and Critical Care Medicine, Keio University School of Medicine, Tokyo 106-8502, Japan; ryoyamamoto@keio.jp; 3 Department of Health Data Science, Graduate School of Data Science, Yokohama City University, Yokohama 236-0004, Japan; 4 Department of Emergency and Critical Care Medicine, St. Marianna University School of Medicine, Kawasaki 216-8511, Japan; 5 Emergency Center, Tohoku University Hospital, Sendai 980-0872, Japan; takesato@hkg.odn.ne.jp; 6 Department of Pharmacy, Kanazawa Medical University Hospital, Kanazawa 920-0293, Japan; yoshi-n@kanazawa-med.ac.jp; 7 Department of Anesthesia and Critical Care, Kobe City Medical Center General Hospital, Kobe 650-0047, Japan; phyandeth69boo@gmail.com (J.I.); gashibe@gmail.com (N.H.); 8 Department of Emergency and Critical Care Medicine, University of Tsukuba Hospital, Ibaraki 305-8576, Japan; nagatomo.k0724@gmail.com; 9 Department of Emergency and Critical Care Medicine, Tohoku University Hospital, Sendai 980-8575, Japan; hohbey@gmail.com; 10 Department of Nephrology, Sapporo Hokushin Hospital, Sapporo 004-8618, Japan; kanac-tak@nifty.com; 11 Department of Nutrition, NHO Kumamoto Medical Center, Kumamoto 860-0008, Japan; kaku.masayuki.az@mail.hosp.go.jp; 12 Department of Acute Care Nursing, Japanese Red Cross Kyushu International College of Nursing, Munakata 811-4157, Japan; gongehead@yahoo.co.jp; 13 Department of Disaster and Emergency Medicine, Graduate School of Medicine, Kobe University, Kobe 650-0017, Japan; nobuto_nakanishi@yahoo.co.jp; 14 Department of Critical Care Medicine and Trauma, National Hospital Organization Disaster Medical Center, Tokyo 190-0014, Japan; fap_kaz09@yahoo.co.jp; 15 Department of Emergency and Critical Care Medicine, Osaka Medical and Pharmaceutical University, Osaka 569-8686, Japan; junji.hatakeyama@ompu.ac.jp; 16 Department of Nursing, Daido Hospital Kojunkai, Social Medical Corporation, Nagoya 457-8511, Japan; amhp_0610@yahoo.co.jp; 17 Department of Emergency Medicine, Fukui Prefectural Hospital, Fukui 910-8526, Japan; fukuiben17@yahoo.co.jp; 18 Department of Preventive Services, Graduate School of Medicine, Kyoto University, Kyoto 606-8501, Japan; tsunemitsu0730@yahoo.co.jp; 19 Department of Intensive Care Medicine, School of Medicine, Sapporo Medical University, Sapporo 060-8543, Japan; tatsumi@qc4.so-net.ne.jp; 20 Department of Critical Care Medicine, Yokohama City University Hospital, Yokohama 236-0004, Japan; knakamura-tky@umin.ac.jp

**Keywords:** enteral feeding intolerance, gastrointestinal complications, nutritional therapy, intensive care unit

## Abstract

Enteral nutrition (EN) has been reported to have some physiological importance for critically ill patients. However, the advantage of EN over parenteral nutrition remains controversial in recent paradigms. To maximize the benefits and efficiency of EN, implementing measures based on comprehensive evidence is essential. Here, we systematically reviewed EN-related studies and integrated them into the best and most up-to-date EN practices. We extracted studies from 13 systematic reviews during the development of Japanese Critical Care Nutrition Guidelines, summarizing findings on the assessment of enteral feeding intolerance (EFI), the timing of EN, formula composition and nutrients, and method of administration in critically ill adult patients. Multifaceted EFI assessment may be needed in patients for high-risk patients. Early EN may reduce infectious complications, and initiating EN even earlier may offer an additional advantage. High protein intake (≥1.2 g/kg/day) could maintain muscle mass and physical function without increasing gastrointestinal complications. Probiotics, prebiotics, and synbiotics may serve as beneficial options for preventing infection and gastrointestinal complications, although their efficacy depends on the strains, types, and combinations used. For patients with EFI, post-pyloric feeding could be an effective approach, while intermittent feeding may be a safer approach. Both methods should be utilized to achieve nutritional targets. Integrating these nutritional interventions into EN strategies may help maximize their effectiveness and minimize complications. However, careful consideration regarding timing, dosage, nutrient selection, administration methods, and patient selection is required.

## 1. Introduction

Nutritional therapy is an essential supportive treatment for all critically ill patients, and a wide variety of interventions—such as early enteral nutrition (EN), appropriate energy and protein administration, and pharmacological nutrients [1,2]—has been investigated in randomized controlled trials (RCTs) and systematic review (SR)/meta-analyses (MA). Some academic organizations have also statistically gathered high-quality evidence and published clinical practice guidelines, which help busy healthcare providers conduct appropriate nutritional therapy for critically ill patients [1,2]. However, due to a lack of evidence, most key clinical questions remain unanswered or yield different recommendations, even in EN, which has been relatively well studied and compared in previous studies. Some guidelines recommend early EN to prevent infectious complications [1,3,4,5], whereas the latest American Society for Parenteral and Enteral Nutrition (ASPEN) guidelines suggest that EN is not superior to parenteral nutrition (PN) [2].

Along with the discrepancy between guidelines, these recommendations may not always be suitable for individual cases or specific clinical situations, as they are developed based on a broad population of critically ill patients following the GRADE system. To optimize the nutritional therapy of EN for each patient, various factors, such as optimal candidates, timing of EN initiation, choice of nutritional formula, and administration route (e.g., gastric vs. duodenal and intermittent vs. continuous), should be carefully considered. Furthermore, the physical outcomes, such as muscle mass and physical function, as well as EN-related complications, should not be overlooked when assessing the EN efficacy and maximizing its utilization.

Accordingly, this narrative review focused on EN and aimed to bridge the gap between clinical practice and guideline recommendations by evaluating a wide range of studies included in the SRs for the Japanese Critical Care Nutrition Guidelines (JCCNG) 2024, considering patient characteristics and clinical contexts, and incorporating all available updated evidence published after the SRs in JCCNG 2024. JCCNG 2024 is unique, incorporating physical function, muscle mass and EN-related complications as essential outcomes in all SRs. A comprehensive and extensive search for studies on EN would identify the best practice of EN for patient-oriented outcomes and help to determine future research areas.

## 2. Materials and Methods

This narrative review was conducted by 20 members involved in the development of the JCCNG 2024 as experts in EN. In JCCNG 2024, 20 SRs were conducted exclusively on RCTs related to nutritional interventions for adult critically ill patients, based on clinical questions (CQ) that the JCCNG Committee deemed clinically important. These CQs were structured using the Patients, Intervention, Control, and Outcome (PICO) framework. In the PICO framework, the study population targeted all critically ill adult patients admitted to the intensive care unit (ICU). The intervention was defined as implementing a specified nutritional therapy, while the control was determined as either the absence of that nutritional therapy or the provision of standard nutritional therapy. Among 20 SRs, we included all 13 SRs related to the implementation of EN for assessment in this review (Table 1, upper section). We discussed EN-related key interventions not covered by SRs (Table 1, lower section) among the narrative review members and conducted hand searches for relevant RCTs.

All SRs addressing nutritional interventions in this guideline were conducted by teams of three to five members in accordance with the Preferred Reporting Items for Systematic reviews and Meta-analysis (PRISMA) 2020 statement [6]. Each systematic team developed tailored search strategies specific to each CQ. Eligible RCTs were sought systematically through MEDLINE (via PubMed), the Cochrane Central Register of Controlled Trials (CENTRAL), and the Igaku Chuo Zasshi (ICHUSHI) database, in April 2023. After ensuring the predefined essential articles were appropriately included, the systematic review team finalized the search strategy. After removing duplicate references, two or more members of the systematic review team independently conducted the primary screening. They used Rayyan (https://rayyan.qcri.org/welcome (accessed on 22 February 2025)) to evaluate titles and abstracts, selecting references that met the PICO criteria (as detailed in the JCCNG 2024 additional file) [7]. The exclusion criteria applied during the primary and secondary screening included the absence of an RCT, an incorrect intervention or control, a language other than English or Japanese, a study population that did not involve critically ill adult patients, or the study being a conference abstract.

For the secondary screening, two or more team members independently reviewed the full text of the selected articles to assess their eligibility based on study design and PICO criteria (as detailed in the JCCNG 2024 additional file). If disagreements arose, a third team member resolved them through discussion. They recorded the reasons for exclusion at each step and summarized them in a PRISMA flow diagram [6].

From the remaining articles, the narrative review team members extracted RCTs related to EN. For interventions covered by SRs, studies published prior to October 2025 were included using the predefined search strategy. For interventions not covered by SRs, designated team members conducted a hand search for RCTs published prior to October 2025 using MEDLINE (via PubMed). Each team member reviewed these studies and summarized key RCTs relevant to EN, focusing on nutritional interventions that were statistically or clinically beneficial or harmful. However, to ensure transparency and minimize bias, nutritional interventions recommended in JCCNG 2024 based on the GRADE methodology were prioritized, particularly those with a large effect size and a moderate or higher certainty of evidence. We also referred to the risk of bias assessments conducted in the guideline development process for individual RCTs. Additionally, we prioritized the inclusion of high-quality, large-scale studies. We described the limitations of studies reporting significant benefits, but with a high risk of bias or small sample sizes. Based on these comprehensive and updated search results, along with the recommendations from JCCNG 2024, the core members of the narrative review team (KK, RY, MY, NH, and KN) determined the best practices for EN through in-depth discussions. This approach enabled a thorough examination and interpretation of the findings, offering insight to optimize the effectiveness of EN, while minimizing its complications in clinical practice.

## 3. Results

### 3.1. Who Can Manage EN with the Fewest Complications?

First, we will discuss who should receive EN to administer it effectively. To carry this out, we must identify which patients are suitable for EN. An SR revealed no large-scale RCTs on this topic [7,8]. However, it is essential to focus on enteral feeding intolerance (EFI) and ventilator-associated pneumonia (VAP), which will be discussed based on previous reports. EFI is a common complication in critically ill patients receiving EN, with an incidence ranging widely from 2% to 75%, depending on the definition and observation method [9,10,11]. EFI is characterized by symptoms such as increased gastric residual volume (GRV), nausea, vomiting, severe diarrhea, abdominal distension, and gastric discomfort [12,13,14,15]. EFI results from impaired digestion and absorption caused by factors such as shock, intestinal edema, and the use of sedatives [16,17,18]. These symptoms are associated with higher mortality, prolonged ICU stays, and an increased risk of infection. GRV monitoring is widely used to evaluate EFI; however, it has limitations [3,19,20]. GRV is affected by patient positioning, feeding tube characteristics, and measurement intervals [21,22]. Therefore, to evaluate GRV, a multidimensional assessment that includes imaging and biochemical markers, such as lactate levels, is essential. Guidelines recommend delaying EN only when GRV exceeds 500 mL/6 h [3]. Although GRV monitoring may reduce the risk of vomiting, it has not been shown to improve mortality, pneumonia incidence, or length of hospital stay [23,24,25]. These findings suggest that GRV monitoring may be particularly beneficial for patients at high risk of aspiration due to vomiting, such as non-intubated patients with disorientation. When GRV is high, the use of prokinetic drugs and continuous enteral feeding rather than intermittent feeding is preferable as a treatment strategy [3,26,27].

Next, we will discuss VAP, which accounts for 80% of hospital-acquired pneumonia in ICU patients [28]; aspiration is a significant concern. Preventive measures include repositioning, enteral feeding tube placement, and continuous EN. Elevating the head of the bed to 30–45 degrees significantly reduced the risk of pneumonia [29]. For patients at high risk of pneumonia, placing enteral feeding tubes beyond the pylorus is recommended [7,30]. Pharmacological interventions, such as synbiotics and semi-solid EN, have shown potential advantages in reducing VAP and gastrointestinal reflux [31]. Physical therapy, such as abdominal massage, has shown potential benefits in reducing gastric content, improving stool consistency, and decreasing VAP incidence [32]. While acupuncture may also help to reduce gastric content in mechanically ventilated patients, its effectiveness against pneumonia remains unclear [33].

Another point to note is that the most serious complication of EN is intestinal ischemia, which requires prompt detection and intervention. Although various biomarkers and imaging modalities are being investigated, the diagnosis remains challenging. A cautious approach, including continuous administration at a small dose, is desirable for at-risk patients, using the available diagnostic tools at each facility (see the Section 3.4) [34,35,36].

### 3.2. When Should EN Be Administered to Be Effective?

Next, we will discuss the optimal timing of EN administration. For critically ill patients unable to maintain voluntary intake, current guidelines recommend early EN within 48 h compared with delayed EN due to a reduction in infection complications [3,7]. Conversely, the ASPEN guidelines suggest that early EN is not superior to early PN when the energy intake is equivalent [26]. Although guideline recommendations vary, recent RCTs have provided further insight into the benefits of early EN and the timing of induction [1,26]. Sun JK et al. evaluated the effects of early EN on immune function in 53 patients with sepsis [37]. When early EN was initiated within 48 h, the proportion of Th17 cells—major mediators of inflammation—decreased, along with the Th17/Treg ratio and inflammatory cytokines IL-17 and IL-23, leading to improved immune balance. These changes suggest that early EN helps to regulate inflammatory responses and restore immune homeostasis, which may contribute to reducing infection complications. Furthermore, early EN shortened ICU stays and the duration of mechanical ventilation. Another RCT, the CALORIES trial, revealed that costs were lower in the early EN group than in the early PN group, despite no significant differences in mortality or infectious complications [38]. Supporting these findings, recent large-scale real-world studies have found that early EN is linked to improved survival, increased discharge home, shorter hospital and ICU stays, reduced mechanical ventilation durations, and lower costs than delayed EN [39]. These studies indicated that early EN might improve outcomes while offering a more cost-effective option.

Furthermore, some studies have suggested that initiating EN even earlier than 24–48 h may provide additional benefits [40,41]. Yu A et al. investigated the efficacy of very early EN (earlier than 24 h) initiation of feeding in 87 critically ill patients [40]. In this study, the intervention group received nasojejunal tube feeding within 6 h of admission, while the control group started nasogastric feeding between 24 and 48 h after admission. The very early EN group improved nutritional status, as evidenced by increased serum albumin and prealbumin levels, enhanced tolerance to nutritional intake, and reduced gastrointestinal and infectious complications.

### 3.3. What Should Be Administered to Effectively Administer EN?

#### 3.3.1. Protein Dose and Type

In the nutritional therapy of critically ill patients, protein administration is an extremely important factor in preventing muscle atrophy and promoting physical function [7,42]. A number of RCTs have examined protein or amino acid administration in EN. Here, we will discuss strategies for optimizing protein delivery in EN.

Critically ill patients often experience impaired protein absorption in their intestinal tracts [43]. However, several RCTs have investigated high protein administration with EN, showing improvements in physical function and muscle mass compared with lower protein intake without an increase in gastrointestinal complications [44,45]. Additionally, studies have reported that, in patients with acute kidney injury (AKI) and severe illness (Sequential Organ Failure Assessment [SOFA] score ≥ 9), high-dose protein intake (1.6 g/kg/day) is associated with increased 60-day mortality compared with normal protein intake (0.9 g/kg/day), without increasing the incidence of diarrhea or vomiting [45]. Given the various modifications to high-protein formulas, they may be feasible even for EFI [44,45]. One approach to preventing EFI is the use of oligomeric or elemental diets consisting of amino acids or small peptides, which are expected to be more easily digested in EN administration [46]. However, the efficacy of oligomeric or elemental diets in preventing EFI in critically ill patients remains uncertain. One study found that an oligomeric formula reduced diarrhea and improved EN achievement compared with a polymeric formula in post-esophagectomy patients [47]. Other studies reported no significant differences in gastrointestinal symptoms among peptide-based, semi-elemental, and polymeric formulas [31,48].

As some amino acids provided via EN, such as glutamine and arginine, offer additional immunomodulatory benefits, enteral glutamine serves as a primary energy source for small intestinal cells, supporting intestinal barrier function [49,50]. Given these advantages, the European Society for Clinical Nutrition and Metabolism (ESPEN) guidelines suggest enteral glutamine for patients with burns or trauma [1]. However, recent large-scale RCTs in burn patients have found no significant differences in outcomes, including mortality, infection, and hospital stay or gastrointestinal complications, with enteral glutamine supplementations [51]. EN enriched with immunomodulating nutrients, including glutamine, omega-3 polyunsaturated fatty acids (PUFAs), and antioxidants, did not improve mortality. Nevertheless, in the sub-group of medical critically ill patients, this intervention was associated with worsening long-term mortality [52]. While enteral arginine supplementation supports wound healing [53], it may have adverse effects in septic shock due to its role in nitric oxide production, which can impair vascular function. Notably, RCTs using arginine-containing immune-enhancing EN for sepsis have shown reduced infection and mortality in mild cases, but increased ICU mortality in severe cases [54,55].

#### 3.3.2. Fat Dose and Type

Omega-3 PUFAs are thought to inhibit the production of inflammatory eicosanoids; hence, these nutrients contribute to the resolution of inflammation [56]. Several RCTs have investigated the efficacy of omega-3 PUFA-enriched EN in critically ill patients with severe acute respiratory failure, sepsis, and trauma [52,57,58,59,60,61,62,63,64,65]. While some studies with EN showed benefits in reducing ICU stay, mechanical ventilation duration and delirium [57,58,59,60,62,64,65], others have reported no benefits in these outcomes [52,59,63,65]. Overall, SR/MA conducted according to the guidelines suggested that omega-3 PUFA may improve ventilation-related outcomes [1,7,66]. Notably, high doses of omega-3 PUFAs (approximately 10 g/day of docosahexaenoic acid plus eicosapentaenoic acid) administered as a bolus were associated with increased mortality, prolonged mechanical ventilation, and extended organ failure duration [63]. When administering omega-3 PUFAs, diarrhea is one of the most frequent adverse events, occurring in 10–28.7% of cases [67]. However, it is generally well tolerated without serious complications [67].

High-fat, low-carbohydrate content via EN is expected to reduce CO_2_ production [68], as fat has the lowest respiratory quotient among the three major nutrient groups [69,70,71]. Additionally, a high-fat, low-carbohydrate content via EN is expected to suppress rapid fluctuations in blood glucose levels and facilitate easier blood glucose management [72,73]. Conversely, the combination of malabsorption, increased bile acid secretion, an imbalance in the gut microbiota, and a rapid transit time caused by a high-fat diet may overwhelm the digestive system’s ability to properly absorb nutrients and fluids, leading to diarrhea [74]. In RCTs on high-fat EN for patients who had undergone mechanical ventilation, no statistically significant differences were observed in mortality, ICU mortality, or ICU length of stay in any of the studies [75,76,77,78,79,80,81,82]. However, two studies reported a reduction in the duration of mechanical ventilation [75,76]. Complications such as diarrhea and GRV were evaluated, and no difference was found between the two groups in any of the studies [77,79,82].

#### 3.3.3. Prebiotics, Probiotics, and Synbiotics

In ICU patients, the intestinal microbiota is drastically altered by illness severity and various medications [83]. Although obligate anaerobes, which normally reside in the human intestinal tract, have a function that suppresses the growth of pathogenic bacteria, called colonization resistance, the number of obligate anaerobes is reduced in ICU patients [84,85]. Pre-/pro-/synbiotics are expected to be effective against this dysbiosis, improving gut microbiota balance and potentially contributing to the prevention of infection and gastrointestinal complications [1]. Prebiotics are a source of nutrients for beneficial bacteria that live symbiotically in the gut. Moreover, probiotics have various effects, including immunomodulation, protection against physiological stress, suppression of pathogenic microbial overgrowth, increased short-chain fatty acids in the intestinal tract, and suppression of intestinal epithelial cell damage [86].

While the 2016 ASPEN guidelines did not recommend routine administration of probiotics for ICU patients [26], a recent SR/MA revealed that probiotics were associated with improvements in VAP [87]. Despite their potential benefits for gut microbiota, no RCT demonstrated a significant improvement in diarrhea (Supplementary Table S1) [88,89,90,91,92,93,94]. Notably, studies that reported beneficial effects used probiotics containing multiple strains with varying combinations [90,94,95]. Conversely, among the eight RCTs administering either a single probiotic or a combination of two strains, only one study using *Clostridium butyricum* demonstrated a significant improvement in constipation [93], while no significant differences were observed for other outcomes [89,91,92,96,97,98,99]. A recent large-scale RCT comparing single-strain probiotics (*Lactobacillus rhamnosus* GG) with a placebo in 2653 ventilated patients found no reduction in the incidence of VAP or diarrhea [92]. Furthermore, the probiotics group experienced significantly more serious infections, with *Lactobacillus* species identical to the administered probiotics detected in sterile sites. An SR of case reports and case series on infectious complications following probiotic intake found that *Saccharomyces cerevisiae* was the most frequently associated pathogen in cases of fungemia, followed by *Lactobacillus rhamnosus* in cases of bacteremia [87]. Additionally, a retrospective study in Japan observed bacteremia caused by *Clostridium butyricum* in immunocompromised patients [100]. Several RCTs on prebiotics in patients with mechanical ventilation found that dietary fiber, particularly soluble fiber, significantly reduced diarrhea and increased EN delivery [101,102,103,104].

An SR/MA revealed an RCT on synbiotics for sepsis that found that synbiotic administration significantly reduced infectious complications and enterocolitis [105]. In a separate RCT, synbiotics may also have the potential to prevent muscle mass loss [106]. Another SR/MA in abdominal surgery revealed that, while synbiotics showed a strong benefit for infection complications, not all synbiotics exhibited favorable trends [107]. A small-sized RCT also demonstrated that preoperative administration of synbiotics in patients undergoing highly invasive surgery for bile duct cancer improved gut microbiota and helped to prevent infectious complications compared with EN only [108]. Moreover, the administration of synbiotics two weeks before surgery improved inflammation and infection complications compared with post-operative administration [109].

### 3.4. How Should EN Be Administered to Patients with Hemodynamic Instability?

Another critical consideration when determining appropriate candidates for EN is hemodynamic instability. Two RCTs have investigated the effect of EN in critically ill patients with hemodynamic instability [34,35]. The characteristics of participants in these trials varied considerably, particularly regarding the required dose of vasoactive agents for hemodynamic stabilization and the administered dose of EN. The NUTRIREA-2 trial [34] compared EN with PN in patients requiring vasoactive agents for shock, with a mean norepinephrine dose of approximately 0.5 µg/kg/min. In the EN group, the mean daily calorie intake was approximately 20 kcal/kg/day, 1 or 2 days after ICU admission, with target calories at 20–25 kcal/kg/day. While there was no difference in mortality between the two groups, gastrointestinal complications—including vomiting, diarrhea, intestinal ischemia, and acute colonic pseudo-obstruction—were significantly more frequent in the EN group. Moreover, a post hoc analysis of the NUTRIREA-2 trial revealed that the use of dobutamine was associated with a threefold higher incidence of intestinal ischemia compared with the non-dobutamine group [110]. Additionally, real-world, large-scale data showed that in patients treated with dobutamine, the association between very early EN initiated the day after cardiac surgery, and outcomes disappeared compared with early EN [41]. However, an ancillary study of the NUTRIREA-2 trial reported that plasma citrulline concentration was higher in the EN group than the PN group, suggesting the possibility that early EN may promote a more rapid restoration of enterocyte mass [111]. Another RCT [35] in patients with septic shock requiring norepinephrine at 0.08 µg/kg/min compared early EN (initiated within 24–48 h of ICU admission) with non-EN. The early EN group started at 20 mL/h (<600 kcal/day) and had significantly more ICU-free and ventilator-free days than the non-EN group, without an increase in any gastrointestinal complications [35].

### 3.5. How Should Enteral Nutrition Be Administered for Maximum Effect?

Finally, we will discuss two main aspects of how EN should be administered: the position of the enteral feeding tube and the method of administration. In critically ill patients, post-pyloric EN is expected to reduce the risk of aspiration [112]. However, the insertion technique and operator skill level may delay the initiation of EN, making it essential to evaluate whether gastric or post-pyloric EN is superior overall. Gastric paresis is common in critically ill patients [113] and often leads to vomiting and aspiration of gastric contents [114]. While gastroprokinetic agents have been proposed for treating gastric paresis [3], studies found that these drugs did not significantly affect the prognosis of critically ill patients [114]. An RCT of 141 patients requiring mechanical ventilation and post-pyloric feeding significantly reduced vomiting (OR 0.30, 95% CI 0.14–0.65) and pneumonia (OR 0.38, 95% CI 0.15–0.94) without differences in mortality [115]. Another RCT involving 70 ICU patients on mechanical ventilation also found that post-pyloric administration significantly reduced VAP, although it did not affect mortality or ICU length of stay [116]. Conversely, placing a feeding tube beyond the pyloric ring requires specialized equipment and expertise, such as endoscopic or fluoroscopic guidance, which may not be available in all facilities [117]. Additionally, post-pyloric feeding tubes are generally more expensive, limiting their routine use. Nevertheless, post-pyloric feeding remains a highly effective method for preventing pneumonia and vomiting, despite these various limitations. It should be actively considered in patients with impaired gastric mobility or at high risk of pneumonia.

Recent RCTs comparing continuous and intermittent EN administration found no significant differences in mortality, duration of mechanical ventilation, incidence of aspiration or gastrointestinal intolerance, including diarrhea and vomiting [118,119,120]. Additionally, an RCT comparing continuous and intermittent feeding in mechanically ventilated patients found no significant difference in nutrition intake or rectus femoris muscle mass change after 10 days in the ICU [118]. Regarding blood glucose levels, intermittent feeding was associated with significantly greater fluctuations compared with continuous feeding [120]. Reports on the achievement of nutritional goals in critically ill patients using continuous versus intermittent feeding strategies have been inconsistent. In one study, intermittent feeding resulted in higher odds of achieving more than 80% of the target protein and energy requirements [118]. Conversely, another study found that, in the continuous enteral feeding group, the same target was achieved significantly more frequently than in the intermittent group [119]. These discrepancies may be attributed to factors such as nutrient composition, administration rate, intervals between intermittent feeds, patient positioning, and the management of GRV (e.g., adjusting infusion rates or using metoclopramide). These studies exclusively targeted patients without EFI [118,119].

## 4. Discussion

This narrative review builds on the 13 SRs conducted during the development of JCCNG 2024, incorporating a comprehensive literature review to identify the best current practices for maximizing the efficacy of EN while minimizing complications. Table 2 provides an overview of each section’s key findings and interpretations. Here, we discuss the summaries of results, interpretations, future research for each intervention, exploring how these findings can be applied to clinical practice, and limitations.

### 4.1. Summary of Results, Interpretations, and Future Research for Each Intervention

Routine GRV measurement may not be necessary for all ICU patients [25]. However, GRV monitoring may be beneficial for patients at high risk of aspiration due to vomiting, such as non-intubated patients with disorientation [23,24,25]. A risk-based, multifaceted EFI assessment—including GRV monitoring—may improve EN-related complications when integrated with comprehensive strategies such as continuous EN, optimal tube placement, and evidence-based physical and pharmacological interventions [3,26,27]. Further research is needed to refine these approaches and establish standardized protocols for the prevention of EFI and EN-related complications.

Early EN demonstrated potential benefits in regulating inflammatory responses and restoring immune homeostasis, which may contribute to preventing infection complications, improving nutritional status, and enhancing cost-effectiveness [39]. These results were derived from studies that included only patients for whom EN was feasible. Moreover, the initiation of very early EN may offer an additional advantage [40,41]. However, evidence on very early EN was limited due to the lack of studies and the small sample sizes. Therefore, the feasibility of EN should be assessed, including factors such as EFI and catecholamine dose, before implementing early or very early EN.

To optimize protein administration via EN, high protein intake in EN of 1.2 g/kg/day or more could improve muscle mass and physical function without gastrointestinal complications [7]. In cases of AKI or severe illness, high protein intake should be avoided during the acute phase [121]. Depending on patients’ condition, protein administration in hydrolyzed form, such as in oligomeric or elemental diets, may reduce gastrointestinal complications and facilitate nutrition delivery [47]. While enteral administration of glutamine and arginine may exhibit immunomodulatory effects, their benefit in critically ill patients appears to be limited [7]. Moreover, in medically severe cases, they may even be harmful, necessitating cautious administration [54,55].

Omega-3 PUFA-enriched EN may be beneficial for mechanically ventilated patients; however, bolus and high doses of administration should be avoided [1,7]. High-fat, low-carbohydrate administration via EN may be preferable to carbohydrate-dependent administration from the perspective of patients on mechanical ventilator support and blood glucose control [122]. The appropriate dosage and ratio remain unclear in these interventions; further research is needed to determine these factors.

Pro-, pre-, and synbiotics are important strategies for preventing infectious complications and maintaining gut homeostasis [7]. However, their efficacy varies depending on the strain of probiotics, type of prebiotics and combination of synbiotics used. Additionally, caution is required when administering certain probiotics to critically ill patients, as they often have preexisting immunosuppression or develop it during their critical illness, potentially increasing the risk of systemic infections [87,92]. Prebiotics, particularly soluble fiber, could effectively reduce diarrhea and achieve EN targets [7]. Synbiotics could reduce infections and may also have the potential to prevent muscle mass loss [7,106]. Synbiotics may be effective not only for critically ill patients requiring emergency admission but also for patients undergoing highly invasive elective surgery [108]. Furthermore, although based on studies with small sample sizes, preoperative synbiotic administration starting two weeks before highly invasive surgery may provide additional benefits [109]. When considering pro-/pre-/synbiotics for critically ill patients, carefully selecting strains and their combinations is essential. Further studies are needed to evaluate the efficacy of individual probiotic strains, specific fiber types, and particular combinations. In addition to gastrointestinal surgery, research focusing on patients undergoing elective cardiovascular surgery is also necessary.

For patients receiving high-dose catecholamines, full feeding via EN should be avoided [34]. Conversely, starting with a small amount of nutrition for patients receiving low-dose catecholamines may be a feasible approach [35], although evidence is limited due to the small number of studies and sample sizes. Additionally, more caution is required when administering EN to patients receiving concomitant dobutamine. Early EN may not be needed to be initiated aggressively in patients receiving dobutamine [41,110]. However, early EN may contribute to intestinal tissue recovery, and it is necessary to assess the dose of catecholamine and EFI daily to determine the timing of initiation of EN [111].

Post-pyloric feeding has clear advantages in reducing vomiting and pneumonia [7]. Although it cannot apply to all critically ill patients or facilities, it should be considered whenever possible, particularly for patients with gastrointestinal intolerance or a high risk of pneumonia. At present, the optimal choice between intermittent or continuous EN for achieving target nutrition remains controversial. However, in cases requiring catecholamine support is required, or gastrointestinal intolerance is present, continuous enteral feeding may be a safer approach, allowing for symptoms and condition monitoring while managing intolerance [35,110,123]. There are few studies investigating the effects of the enteral feeding tube position or the method of administration on physical function and muscle mass. Further research is needed to assess their impact on these outcomes.

### 4.2. How the Obtained Findings Can Be Applied to Clinical Practice

**Figure 1:**
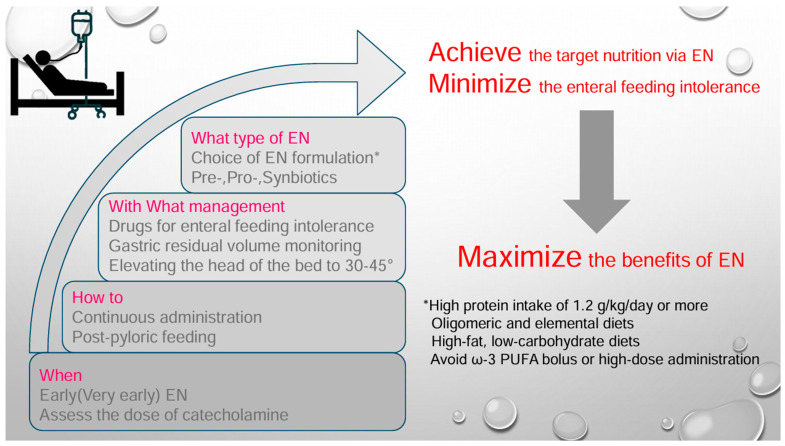
This key diagram summarizes essential strategies for minimizing enteral feeding intolerance and achieving target EN delivery. Priorities are arranged in descending order from top to bottom. EN, enteral nutrition; PUFAs, polyunsaturated fatty acids.

Early EN, and if feasible, very early EN, is advisable while carefully assessing gastrointestinal tolerance and catecholamine dosage. Initially, continuous feeding is preferred, gradually increasing to achieve adequate energy and protein intake. In cases of high GRV or vomiting, post-pyloric feeding is a favorable option to reduce the incidence of VAP and improve nutritional goal achievement. If post-pyloric feeding is unavailable, pharmacological interventions could be considered alternative options. Incorporating prebiotics and synbiotics with EN may be beneficial for minimizing infectious and gastrointestinal complications while achieving target nutritional intake (Figure 1).

Various nutritional interventions need to be incorporated to maximize the benefits of EN. Ongoing research on protein dosage [124,125], omega-3 PUFA fatty acid supplementation [126], and fasting intervals [127,128,129] are expected to refine EN strategies further, enabling more effective implementation in clinical practice.

### 4.3. Limitations

This narrative review summarizes the results of RCTs on EN-related nutritional interventions in a broad population of critically ill patients. However, heterogeneity exists due to the inclusion of patients with varying disease severities and conditions. Consequently, we could not provide EN strategies tailored to specific diseases and severity levels in this review. In recent years, patient-oriented outcomes, such as quality of life and physical function, have gained increasing attention as key measures of nutritional therapy [130]. However, in this review, few studies have evaluated these outcomes, leaving the impact of EN-related nutritional interventions on patient-centered outcomes unclear. Future RCTs should incorporate patient-oriented outcomes to better assess the benefits of EN-related interventions.

## 5. Conclusions

This review discusses strategies for optimizing EN implementation from multiple perspectives based on the results of RCTs identified through a comprehensive and updated search. EN-related interventions such as early EN, high-protein diets, omega-3 PUFA, prebiotics, synbiotics, and post-pyloric feeding may be beneficial. However, careful consideration regarding timing, dosage, strain selection, nutrient type selection and combination selection, administration methods, and patient selection is required. Integrating these nutritional interventions into EN strategies may help optimize their effectiveness.

To maximize the benefits of EN, it is essential to clarify *who*, *when*, *what type of nutrients*, and *how to* administer; therefore, we summarized realistic practices based on what is currently known for each part. Reducing complications related to EN, including gastrointestinal complications, while implementing these realistic, practical methods, is crucial for efficiently administering EN.

## Figures and Tables

**Table 1:** Search methods for enteral nutrition-related key interventions in this review.

No.	Interventions covered by SRs in JCCNG 2024
1	High-dose protein administration
2	Early EN within 48 h
3	EN in hemodynamically unstable patients
4	Post-pyloric and gastric feeding for EN
5	Continuous and intermittent feeding for gastric EN
6	Omega-3 fatty acid-enriched EN
7	Glutamine-enriched EN
8	Oligomeric, elemental, and polymeric EN
9	Arginine-enriched EN
10	High-fat, low-carbohydrate EN
11	Prebiotics in EN
12	Probiotics in EN
13	Synbiotics in EN
	Interventions not covered by SR (by hand search)
1	Assessment of enteral feeding intolerance
2	Strategies to reduce the risk of aspiration in EN
3	GRV monitoring in EN
4	Intestinal ischemia

EN enteral nutrition; GRV, gastric residual volume; JCCNG, Japanese Critical Care Nutrition Guidelines; SR, systematic review.

**Table 2:** Outline of a large-scale RCT related to EN administration.

Who can manage EN with the fewest complications? GRV monitoring may be considered for patients at risk of aspiration, such as non-intubated patients with impaired consciousness. A multifaceted EFI risk assessment, along with an integrated approach encompassing appropriate tube placement, physical interventions, and pharmacological strategies, may help to prevent and manage EN-related complications.
When should EN be administered to be effective? Early EN, when feasible, may control inflammation and prevent infection complications. Additionally, it appears to be more cost-effective. The initiation of very early EN, rather than early EN within 48 h, may offer an additional advantage.
What should be administered to effectively administer EN? (Protein) A high protein intake of 1.2 g/kg/day or more may maintain muscle mass and improve physical function. Oligomeric and elemental diets may help to reduce gastrointestinal complications and achieve targeted nutritional intake in selected cases. While enteral glutamine and arginine may exhibit immunomodulatory effects, their benefit in critically ill patients appears to be limited. (Fat) Although EN enriched with omega-3 PUFAs may improve mechanical ventilation duration, bolus or high-dose administration should be avoided. For mechanically ventilated patients, high-fat, low-carbohydrate EN may be preferable to carbohydrate-based feeding. (Pre-/pro-/synbiotics) Pre-/pro-/synbiotics would be valuable adjunctive therapies for preventing infectious and gastrointestinal complications while maintaining intestinal homeostasis. Careful consideration is required when selecting strains of probiotics, types of prebiotics, and combinations of synbiotics, as some may be ineffective or lead to severe infections in immunocompromised patients. Administering synbiotics before ICU admission may offer additional advantages, particularly for elective surgery, with initiation ideally starting 2 weeks in advance.
How should EN be administered to patients with hemodynamic instability? For patients receiving high-dose catecholamines, a full dose of EN should be avoided. For patients receiving dobutamine, early EN may not need to be prioritized due to the risk of intestinal ischemia and potential ineffectiveness.
How should enteral nutrition be administered to maximize effect? (Gastric versus Post-pyloric feeding) Post-pyloric feeding should be considered whenever possible in case of gastrointestinal intolerance or for patients at high risk of pneumonia. (Continuous administration vs. Intermittent administration) When gastrointestinal intolerance is present, continuous EN may be a safer approach, allowing for symptoms and condition monitoring while managing intolerance.

EN, enteral nutrition; EFI, enteral feeding intolerance; GRV, gastric residual volume; ICU, intensive care unit; PUFAs, polyunsaturated fatty acids.

## Supplementary Materials

The following supporting information can be downloaded at: https://www.mdpi.com/article/10.3390/nu17050845/s1, Table S1: Summary of randomized controlled trials on probiotics in this narrative review.

## Abbreviations

ASPEN	American Society for Parenteral and Enteral Nutrition
AKI	acute kidney injury
CQ	clinical questions
CRICS	Clinical Research in Intensive Care and Sepsis
EFI	enteral feeding intolerance
EN	enteral nutrition
ESPEN	European Society for Clinical Nutrition and Metabolism
GRV	gastric residual volume
ICU	intensive care unit
JCCNG	the Japanese Critical Care Nutrition Guidelines
MA	meta-analyses
PICO	patients, intervention, control, and outcome
PN	parenteral nutrition
PRISMA	Preferred Reporting Items for Systematic reviews and Meta-analysis
PUFAs	polyunsaturated fatty acids
RCT	randomized control trial
SCCM	Society of Critical Care Medicine
SOFA	Sequential Organ Failure Assessment
SR	systematic review
VAP	ventilator-associated pneumonia
