# Supplementary material for: Strategies to Maximize the Benefits of Evidence-Based Enteral Nutrition: A Narrative Review
Source: Nutrients. 2025 Feb 28;17(5):845. doi: 10.3390/nu17050845 (PMC11901663; doi:10.3390/nu17050845)
Supplement: Supplementary file 1 [file nutrients-17-00845-s001.zip › nutrients-3503320-supplementary.pdf]

Supplementary Table S1. Summary of randomized controlled trials on probiotics in this narrative review

Author	Year	Population	Sample size	Intervention	Control	Findings
Forestier C et al.	2008	Mixed ICU, France<br>Patients aged 18 years or older with a stay longer than 48 hours	N = 208	***Lactobacillus casei rhamnosus*** (1 × 10^9 CFU; the available pharmaceutical form no E01-A02-S06), twice daily from the third day after admission to the ICU until discharge or death	Placebo	VAP: 3 (2.9%) vs. 8 (7.5%), p = NA
Barraud D et al.	2010	Mixed ICU, France<br>Adult patients under mechanical ventilation for a predicted period of at least 2 days	N = 167	Ergyphilus® capsules containing 2 × 10^{10} of revivable bacteria (mainly ***Lactobacillus rhamnosus GG***, ***Lactobacillus casei***, ***Lactobacillus acidophilus***, ***Bifidobacterium bifidum***), once a day the entire period of mechanical ventilation, but for a duration not exceeding 28 days	Placebo	28-day mortality: 21 (24.1%) vs. 21 (26.2%), p = NA<br>28-day mortality: 22 (25.3%) vs. 19 (23.7%), p = 0.80<br>90-day mortality: 27 (31.0%) vs. 24 (30.0%), p = 0.90<br>All ICU-acquired infections: 30 (34.4%) vs. 30 (37.5%), p = NA<br>OR 0.87 (95% CI 0.46–1.65, p = 0.68)<br>Diarrhea: 48 (55.2%) vs. 42 (52.5%), p = NA
Sharma B et al.	2011	The ward of the Department of Gastroenterology, India<br>Patients with acute pancreatitis presenting within the first 72 hours	N = 50	Four sachets of Probiotics (about 2.5 billion bacteria per sachet containing ***Lactobacillus acidophilus***, ***Bifidobacterium longus***, ***Bifidobacterium bifidum***, and ***Bifidobacterium infantalis*** with 25 mg of fructo-oligosaccharide) per day, the duration of intervention was 7 days.	Placebo	Hospital mortality: 2 (8.3%) vs. 2 (7.7%), p = 1.000
Tan M et al.	2011	Surgical ICU, China<br>Closed head injury alone; admission within 24 hours after trauma	N = 52	***Bifidobacterium longum*** (0.5 × 10^8), ***Lactobacillus bulgaricus*** (0.5 × 10^7), and ***Streptococcus thermophilus*** (0.5 × 10^7), three times a day. for 21 consecutive days	Placebo	28-day mortality: 3 (11.5%) vs. 5 (19.2%), p = 0.701<br>VAP: 7 (43.8%) vs. 13 (68.4%), p = 0.182<br>Severe infection: 9 (34.6%) vs. 15 (57.7%), p = 0.095
Juan Zeng et al.	2016	Mixed ICU, multicenter, China<br>All critically ill adult patients (age 18 years and older) with an expected need of mechanical ventilation for at least 48 hours	N = 235	Probiotics capsule contained active ***Bacillus subtilis*** and ***Enterococcus faecalis*** at a concentration of 4.5 × 10^9 CFU and 0.5 × 10^9 CFU, three times daily, until tracheal extubation, discharge from the hospital or	Placebo	ICU mortality: 15 (12.7 %) vs. 9 (7.7 %), p = 0.207<br>Hospital mortality: 11 (10.7 %) vs. 16 (14.8 %), p = 0.369<br>VAP: 48 (40.7%) vs. 62 (53.0%), p = 0.059

				death, with a maximum study duration of 14 days		
Alberdav C et al.	2018	ICU, multicenter, Canada<br>Age > 18 years; prescribed one or more antibiotics in ICU	N = 32	Probiotic yogurt drink, Danactive® (Danone, Boucherville, QC, Canada) containing ***Lactobacillus casei sp. Paracasei CNCM I-1518*** (formally DN-114 001) (1×10^9 CFU), twice daily<br>Once antibiotic therapy was completed, probiotic therapy continued for 7 days and discontinued at the time of ICU discharge	Placebo	ICU mortality: 1 (6.3%) vs. 2 (12.5%), p = 1.000<br>Hospital mortality: 2 (12.5%) vs. 2 (12.5%), p = 1.000<br>30 days mortality: 3 (18.8%) vs. 4 (25%), p = 1.000<br>Diarrhea: 11 (68.8%) vs. 10 (62.5%), p = 1.000
Anandaraj et al.	2019	Medical ICU, India<br>Intubated adults over the age of 18 were screened	N = 146	***Lactobacillus rhamnosus*** (2×10^9 CFU), twice daily, a total of 7 days or until extubation	Placebo	ICU mortality: 22 (31%) vs. 20 (27%), p = 0.64<br>Hospital mortality: 28 (39%) vs. 30 (41%), p = 0.83<br>VAP: 7 (10%) vs. 8 (11%), p = NA<br>Early VAP: 6 (8.3%) vs. 5 (6.8%), p = 0.31
Mahmoodpoor A et al.	2019	Surgical ICU, Iran<br>Adults (>18 years) mechanically ventilated	N = 100	10^{10} bacteria consisting of ***Lactobacillus spp.*** (***casei, acidophilus, rhamnosus, bulgaricus***), ***Bifidobacterium spp.*** (***breve, longum***), and ***Streptococcus thermophilus.***<br>1 capsule every 12 hours for 14 days	Placebo (sterile maize starch powder)	ICU mortality: 5 (10.1%) vs. 6 (11.1%), p = 0.58<br>VAP incidence（as per 1000 hours）: 0.66 vs. 0.94, p = 0.04<br>Diarrhea: 7 (14.4%) vs. 15 (27.8%), p = 0.08
Tamer Habib et al.	2020	Trauma ICU, Egypt<br>Adults (>18 years) mechanically ventilated	N = 65	Lacteol Forte® sachet containing ***Lactobacillus LB*** (1×10^9 CFU of ***Lactobacillus delbruecki***, ***Lactobacillus fermentum***), three times daily, during ICU stay	Placebo	ICU mortality: 11 (34.4%) vs. 12 (36.4%), p = 1.000<br>VAP: 5 (15.6%) vs. 7 (21.2%), p = 0.751
Nazari B et al.	2020	ICU, multicenter, Iran<br>Mechanical ventilation for at least 48 hours	N = 147	***Lactocare*** capsules (Zist Takhmir Company-Tehran-Iran) with 20 cc of distilled water through a nasogastric tube once every 12 hours	Placebo (only	VAP: 9 (12.3%) vs. 33 (44.6%), p = 0.001
Litton E et al.	2021	ICU, multicenter, Australia<br>Adults within 48 hours of ICU admission	N = 218	***L. plantarum 299v*** (2×10^{10} CFU) per capsule during the 60 days	microcrystalline cellulose)	ICU mortality: 4 (3.6%) vs. 4 (3.7%), OR 0.98 (0.24–4.03), p = 1.00<br>Hospital mortality: 5 (4.6%) vs. 4 (3.7%), OR 1.24 (0.32–4.74), p = 1.00

						Nosocomial infection: 8 (7.3%) vs. 5 (4.6%), OR 1.62 (95% CI 0.51–5.1), p = 0.57<br>Probiotic group: diarrhea 1 (0.9%),<br>Placebo group: large bowel obstruction 1 (0.9%)
Johnstone J et al.	2021	ICU, multicenter, Canada, the USA, and Saudi Arabia<br>Adult (> 18 years old) expected to require mechanical ventilation for at least 72 hours	N = 2650	***L rhamnosus GG*** (i-Health Inc)(1×10^{10} CFU), twice daily<br>up to 60 days or until the discharge from the ICU or until Lactobacillus spp. were isolated from a sterile site or cultured as the sole or predominant organism from a nonsterile site	Placebo (sterile maize starch powder)	ICU mortality: 279 (21.2%) vs. 296 (22.2%), HR 0.91 (0.77–1.08), p = 0.30<br>Hospital mortality: 363 (27.5%) vs. 381 (28.6%), HR 0.91 (0.79–1.06), p = 0.21<br>VAP: 289 (21.9%) vs. 284 (21.3%), HR 1.03 (0.87 – 1.22), p = 0.73<br>Diarrhea: 861 (65.3%) vs. 855 (64.2%), HR 1.01 (0.91–1.11), p = 0.90
Tsilika M et al.	2021	ICU, multicenter, Greece<br>Adults aged 18–80 years,<br>recent trauma involving head injury	N = 112	The probiotic preparation (LactoLevure, UniPharma, Athens, Greece) was a combination of four probiotics: ***L. acidophilus LA-5*** (1.75×10^9 CFU), ***L. plantarum UBLP-40*** (0.5×10^9 CFU), ***B. animalis subsp. lactis BB-12*** (1.75×10^9 CFU) and ***S. boulardii Unique-28*** (1.5×10^9 CFU). twice daily, for 15 days	Placebo	30-day mortality: 3 (5.1%) vs. 2 (3.8%), p = 1.00<br>VAP: 7 (11.9%) vs. 15 (28.3%), p = 0.034<br>Central catheter-associated infection: 7 (11.9%) vs. 11 (20.8%), p = 0.303<br>Bloodstream infection: 16 (32.2%) vs. 15 (28.3%), p = 0.685<br>Sepsis: 4 (6.8%) vs. 13 (24.5%), p = 0.016<br>Septic shock: 2 (3.4%) vs. 5 (9.4%), p = 0.253
Wang J et al.	2021	Respiratory intensive care unit, China<br>Age >18-year-old patients newly admitted to respiratory intensive care unit	N = 61	MIYA-BM® tablets (Miyarisan Pharmaceutical Co., Ltd., Tokyo, Japan), contains ***Clostridium butyricum*** at 10^6 CFU bacteria per sachet. three times daily, for 14 days	Placebo	All mortality: 6 (0.21%) vs. 7 (0.21%), p = 0.98<br>Blood infection: 1 (0.04%) vs. 1 (0.03%), p = 0.91<br>Diarrhea: 12 (0.43%) vs. 8 (0.24%), p = 0.12<br>Constipation: 5 (0.05%) vs. 14 (0.13%), p = 0.00
Prasoon A et al.	2022	Trauma ICU, India<br>All critically ill patients of at least 18 years of age who were mechanically ventilated for more than 48 hours.	N = 120	Commercially available probiotics, that is, orodispersible probiotic sachet that consists of ***Lactobacillus acidophilus***, ***Lactobacillus rhamnosus, Bifidobacterium longum***, and	Standard preventive strategies	ICU-associated diarrhea: 0 (0.0%) vs. 4 (8.0%), p = 0.001<br>*Clostridium difficile*-associated diarrhea: 1 (1.92%) vs. 6 (12%), p = 4.0

				Saccharomyces boulardii twice daily		Constipation: 3 (5.7%) vs. 25 (50%), p = 0.001
Tzikos G et al.	2022	Trauma ICU, multicenter, Greece<br>Adult patients with recent trauma involving brain injury and at least one organ failure, requiring urgent intubation and anticipated prolonged mechanical ventilation (≥10 days).	N = 103	***Lactobacillus acidophilus LA-5*** (1.75 × 10^9 CFU)<br>***Lactiplantibacillus plantarum*** (0.5 × 10^9 CFU)<br>***Bifidobacterium lactis BB-12*** (1.75 × 10^9 CFU)<br>***Saccharomyces boulardii*** (1.5 × 10^9 CFU)**,**<br>twice daily, for 15 days	Placebo	SSI: 5 (9.4%) vs. 15 (30%), p<0.001
Lu P et al.	2024	Mixed ICU, China<br>Adult patients (>18 years) expected to require mechanical ventilation for more than 72 hours.	N = 24	Combined ***Bifdobacterium***, ***Lactobacillus*** and ***Enterococcus*** capsules, twice a day, until leaving the ICU	Placebo	28-day mortality: 2 (16.7%) vs. 4 (33.3%), p = 0.346<br>VAP: 0 vs. 1 (8.3%), p = 1.000

Abbreviations: CFU; colony-forming units, HR, hazard ratio; ICU, intensive care unit; NA, not available; OR, odds ratio; SSI, surgical site infection; VAP, ventilator-associated pneumonia.
